# Development of a Nomogram for Peak Nasal Inspiratory Flow Among Indian Children: A Hospital-Based Observational Study

**DOI:** 10.7759/cureus.109948

**Published:** 2026-05-30

**Authors:** Samya Mitra, Nihar R Mishra, Ritesh Singh, Subhankar Sarkar, Aditi Das, Niladri S Bhunia, Rohit Bhowmick, Rimjhim Sonowal

**Affiliations:** 1 Pediatrics, All India Institute of Medical Sciences (AIIMS), Kalyani, IND; 2 Community and Family Medicine, All India Institute of Medical Sciences (AIIMS), Kalyani, IND; 3 Pediatric Critical Care Medicine, All India Institute of Medical Sciences (AIIMS), Kalyani, IND

**Keywords:** allergic rhinitis, clinical nomogram, indian children, peak nasal inspiratory flow, pediatric allergy

## Abstract

Objective: This study aimed to develop a nomogram for the peak nasal inspiratory flow (PNIF) meter among Indian children and to determine the cutoff value of peak nasal inspiratory flow to detect allergic rhinitis and the blocker variety, as well as to look for differences in PNIF value among age groups.

Methods: A cross-sectional study was conducted in the pediatric outpatient department of a tertiary hospital among children aged six to 17 years. The PNIF data were obtained along with demographic variables. Children were diagnosed with allergic rhinitis, with or without blockage symptoms, according to the Allergic Rhinitis and its Impact on Asthma (ARIA) classification. A total of 573 children were included after applying the exclusion criteria. Data analysis was done using SPSS Statistics version 25 (IBM Corp., Armonk, NY, USA) and Dxt version 1.0 (Biostatistics Resource and Training Centre, Christian Medical College Vellore, TN, IND).

Results: The mean PNIF value of all age groups was 67.95 L/min (standard deviation 13.46). The PNIF has a statistically significant but weak correlation with age and a statistically significant difference between age groups. Cutoff values for both allergic rhinitis and blocker varieties were 65 L/min, with varying sensitivity and specificity across age groups. Multiple linear regression analysis derived the equation for PNIF = 53.061 + (1.689 × age) + (1.312 × gender) + (-0.015 × BMI).

Conclusion: The PNIF with set cutoff values can be used to diagnose allergic rhinitis and the blocker variety in Indian children. It can be used as a diagnostic tool to determine allergic rhinitis with modest diagnostic accuracy in the six to nine-year and 13 to 17-year age groups. For children more than 10 years of age, PNIF demonstrates clinical utility in determining the obstructive nasal phenotype.

## Introduction

Peak nasal inspiratory flow (PNIF) is a valuable diagnostic tool for assessing nasal airway patency. It is recognized as a quick, user-friendly objective method that directly quantifies nasal airflow during a patient's maximal inspiratory effort [1,2]. This technique provides a straightforward measure of how much air a person can draw through their nasal passages, offering immediate insights into potential obstructions.

The utility of PNIF in identifying nasal obstructive changes is well-documented. Previous clinical studies have shown its accuracy, with reported sensitivity around 0.66 and specificity around 0.80 [3]. This translates to a diagnostic accuracy of approximately 0.757, making it a reliable instrument in differentiating between normal and obstructed nasal breathing [3]. These metrics signify that PNIF can correctly identify a substantial proportion of individuals with genuine nasal obstruction while also effectively ruling out obstruction in those with patent airways. Rhinomanometry has been traditionally established as a modality to quantify nasal obstruction in children [4,5]. Given the limited access to objective diagnostic tools such as rhinomanometry, the PNIF meter offers a practical, cost-efficient alternative.

A commonly referenced threshold for indicating significant nasal obstruction is a PNIF value below 120 L/min [6]. This cutoff point serves as a clinical indicator, suggesting that airflow is compromised to a degree that may warrant further investigation or intervention. Currently, there is a notable lack of robust, population-specific data on typical PINF values for healthy Indian children as well as those with underlying conditions such as allergic rhinitis. 

As per the Allergic Rhinitis and its Impact on Asthma (ARIA) guidelines [7], allergic rhinitis is defined as type 1 hypersensitivity inflammation of the nasal mucosa, induced by exposure to an allergenic substance, having at least two cardinal nasal symptoms: (1) sneezing, rhinorrhea, nasal itching, and (2) nasal block present for more than one hour per day for >2 weeks in a year. According to predominant nasal symptoms, it can be the blocker variety, where predominant nasal symptoms are nasal block or obstruction, or the non-blocker variety, which can be sneezing or rhinorrhea.

While a few previous studies have explored the utility of PNIF as a general objective tool for assessing nasal airway obstruction in children, these studies have often been limited in scope and have primarily focused on validating PNIF against other measures or establishing its feasibility [8-11]. Facial changes associated with mandibular growth and adenotonsillar hypertrophy have been shown to impact the PNIF values [12-14]. Predominant adenotonsillar growth occurs between ages six and nine. Hence, three age groups of six to nine years, 10 to 12 years, and 13 to 17 years were decided for this study. The primary objective of this study is to develop a nomogram for the PNIF among Indian children. The secondary objective was to develop cutoff values for PNIF in children with allergic rhinitis and in those with the blocker variety (the nasal obstruction-predominant subtype as defined by the ARIA classification [7]), as well as to look for any statistical difference in PNIF value between age groups.

## Materials and methods

A cross-sectional study was conducted in the pediatric OPD of All India Institute of Medical Sciences (AIIMS) in Kalyani, West Bengal, India, and approved by its Institutional Ethics Committee (approval no. IEC/AIIMS/Kalyani/Meeting/2023/096). The study included pediatric patients aged 6 to 17 years who attended routine health check-up visits at the primary health center managed by the Department of Community and Family Medicine. These patients were then referred to the pediatric OPD for detailed assessment and PNIF measurement. Patients with acute-onset breathing difficulty or those having chronic lung diseases, chronic heart diseases, congenital anomalies, or chest wall deformities, and those who had been receiving any treatment for allergic rhinitis were excluded. A total of 573 participants were enrolled in the study after obtaining their informed written consent and assent (where applicable).

Sample size estimation was performed using nMaster version 2.0 (Biostatistics Resource and Training Centre (BRTC), Christian Medical College Vellore, TN, IND) to estimate a confidence interval for a single proportion, using the absolute precision method with a finite population correction factor. Population size was taken as n/N = 1,000,000, assuming a prevalence (p) of allergic rhinitis among 24.4% of children with an absolute precision (d) of 4%, a confidence level of 95%, and a design effect (DEFF) of 1. Thus, the following sample size formula was used. \[{n = [DEFF × Np (1-p)] / [(d2/Z21-α/2 × (N-1) +p × (1-p)]}\] The minimum sample size was calculated to be 443. The final minimum sample size was 487 after adjusting for 10% attrition. A 10% attrition adjustment was applied to account for anticipated difficulty in some patients' inability to perform a technically acceptable sniff effort despite coaching, particularly those aged six to seven years.

Patients were screened against the pre-defined inclusion and exclusion criteria. During each visit, up to 30 eligible patients’ consultation booklets were numbered sequentially. Participants were then selected using simple random numbers generated via Microsoft Excel (Microsoft Corp., Redmond, WA, USA), with only patients corresponding to the randomly generated numbers enrolled in the study.

Following participant selection, written informed consent from parents and assent from the pediatric patients (where applicable) were obtained before data collection commenced. Demographic and anthropometric data were gathered, encompassing details such as age, gender, height, weight, and BMI, using a pre-designed case report form. Relevant medical history was taken, and the participants were classified as having allergic rhinitis (with/without blocker symptoms) or not, according to the Allergic Rhinitis and its Impact on Asthma (ARIA) classification [7,15-17]. All case records were coded to maintain confidentiality. Participants were subdivided into three age groups as mentioned earlier.

Nasal airway patency was assessed using the Peak Nasal Inspiratory Flowmeter (GM Instruments Ltd., Irvine, Ayrshire, GBR), used with a pediatric (small) size disposable or reusable mask. The flowmeter comprised a variable-diameter tube calibrated in L/min, along with a low-inertia indicator ring whose position after an inspiratory maneuver (sniffing) clearly indicated the maximum flow achieved. An average reading from the best of three maneuvers was recorded for each participant, allowing for variability in participant effort and the possible effect of nasal valve collapse. An accuracy of ±10% of reading or 10 L/min flow (whichever was greater) with repeatability of ±5% was considered.

Statistical analysis

Data were analyzed using SPSS Statistics version 25.0 (IBM Corp., Armonk, NY, USA). Data normalcy was tested by the Shapiro-Wilk test. All continuous data were expressed as mean and standard deviation; however, categorical data were expressed as proportions. Correlation statistics were computed between continuous variables (age, BMI, and PNIF) with the Spearman correlation coefficient.

To develop a nomogram, a multiple linear regression model was built to predict PNIF using age (in years), gender (categorical), and BMI (continuous). Linearity was assessed by inspection of residual-versus-fitted plots; independence was inherent to the cross-sectional design; homoscedasticity was evaluated using the Breusch-Pagan test [18]; and normality of residuals was confirmed by the Shapiro-Wilk test on model residuals. Variance inflation factors (VIF) were calculated for all predictors; all VIF values were <3, indicating no significant multicollinearity. Gender was coded as a binary variable (male = 1, female = 0); age was entered as a continuous variable in years; BMI was entered as a continuous variable in kg/m². Missing data were minimal (<1% across all variables) and handled by complete-case analysis (listwise deletion).

Multiple linear regression was run with ‘PNIF’ as the dependent variable and 'age,' 'gender,' and ‘BMI’ as the independent variables. The cutoff value of PNIF was estimated using Dxt version 1.0 (BRTC). Validation was performed using the bootstrapping method in SPSS Statistics version 25.0 using 1000 bootstrap samples with bias-corrected and accelerated 95% confidence intervals to assess the stability and internal validity of the regression model. For all statistical purposes, a p-value < 0.05 was considered significant.

## Results

A cohort of 573 pediatric patients with a mean (SD, range) age of 11.23 (3.37, six to 17) years was included in the study. Of these, 201 (35%), 159 (28%), and 213 (37%) patients belonged to the age groups of six to nine years, 10 to 12 years, and 13 to 17 years. Table 1 depicts the baseline characteristics.

**Table 1:** Baseline characteristics of the study population (total n = 573) The values are expressed as n (%) or mean (SD).

Variables	Value
Gender	
Boys	304 (53)
Girls	269 (47)
Anthropometry	
Height (cm)	139.3 (17.6)
Weight (kg)	36.1 (14.5)
Body Mass Index (kg/m2)	17.9 (4.1)
Allergic Rhinitis	323 (56) (95% CI: 52.2%-60.5%)
Six to nine years	98 (30)
10 to 12 years	99 (30)
13 to 17 years	126 (40)
Allergic rhinitis (blocker variety)	119 (47)
Six to nine years	36 (30)
10 to 12 years	53 (44)
13 to 17 years	30 (26)
PNIF (L/min)	67.95 (13.46)
Age-wise PNIF	
Six to nine years	62.44 (11.06)
10 to 12 years	68.76 (14.44)
13 to 17 years	72.56 (12.94)
Gender-wise PNIF	
Male	68.05(12.82)
Female	67.85(14.17)
Allergic rhinitis (n = 323)	65.11 (13.91)
Allergic rhinitis (blocker variety) (n = 119)	64.32 (16.39)

The original value of the mean with SD of PNIF for the group was 67.95 (13.46) L/min. Analysis revealed a weak yet statistically significant positive correlation between age and PNIF values (Spearman's correlation coefficient = 0.370; p < 0.001). The ANOVA indicated significant differences in mean PNIF across age groups; patients aged six to nine years had a significantly lower mean PNIF (62.44 (11.06) L/min) compared to those aged 10 to 12 years (68.76 (14.44) L/min, p < 0.001) and those aged 13 to 17 years (72.56 (12.95) L/min, p < 0.001).

**Figure 1:**
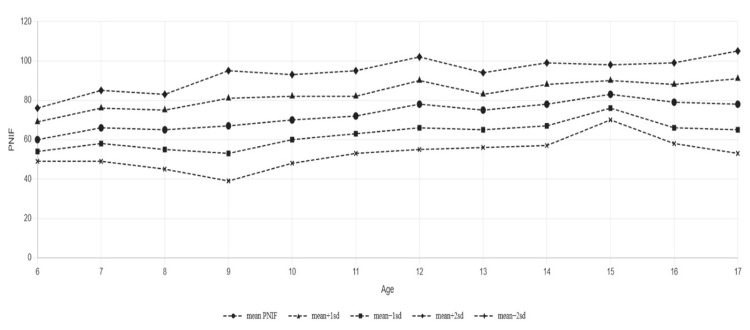
Nomogram of PNIF across age groups PNIF: Peak nasal inspiratory flow

No statistically significant difference was found in the mean (SD) PNIF values of boys (68.05 (12.83)) and girls (67.85 (14.17); p = 0.859). The BMI exhibited a weak but significant positive correlation with PNIF (Spearman's coefficient = 0.167; p < 0.001). A clinical nomogram was developed based on age groups and original PNIF values as depicted in Figure 1. Multiple linear regression produced the following predictive equation for PNIF: PNIF = 53.061 + (1.689 × age) + (1.312 × gender) + (-0.015 × BMI). In this model, age was the only statistically significant predictor of PNIF, whereas gender and BMI were not significant. The model’s relatively low adjusted R2 (0.230) underscores the presence of other unmeasured factors contributing to PNIF variability.

**Figure 2:**
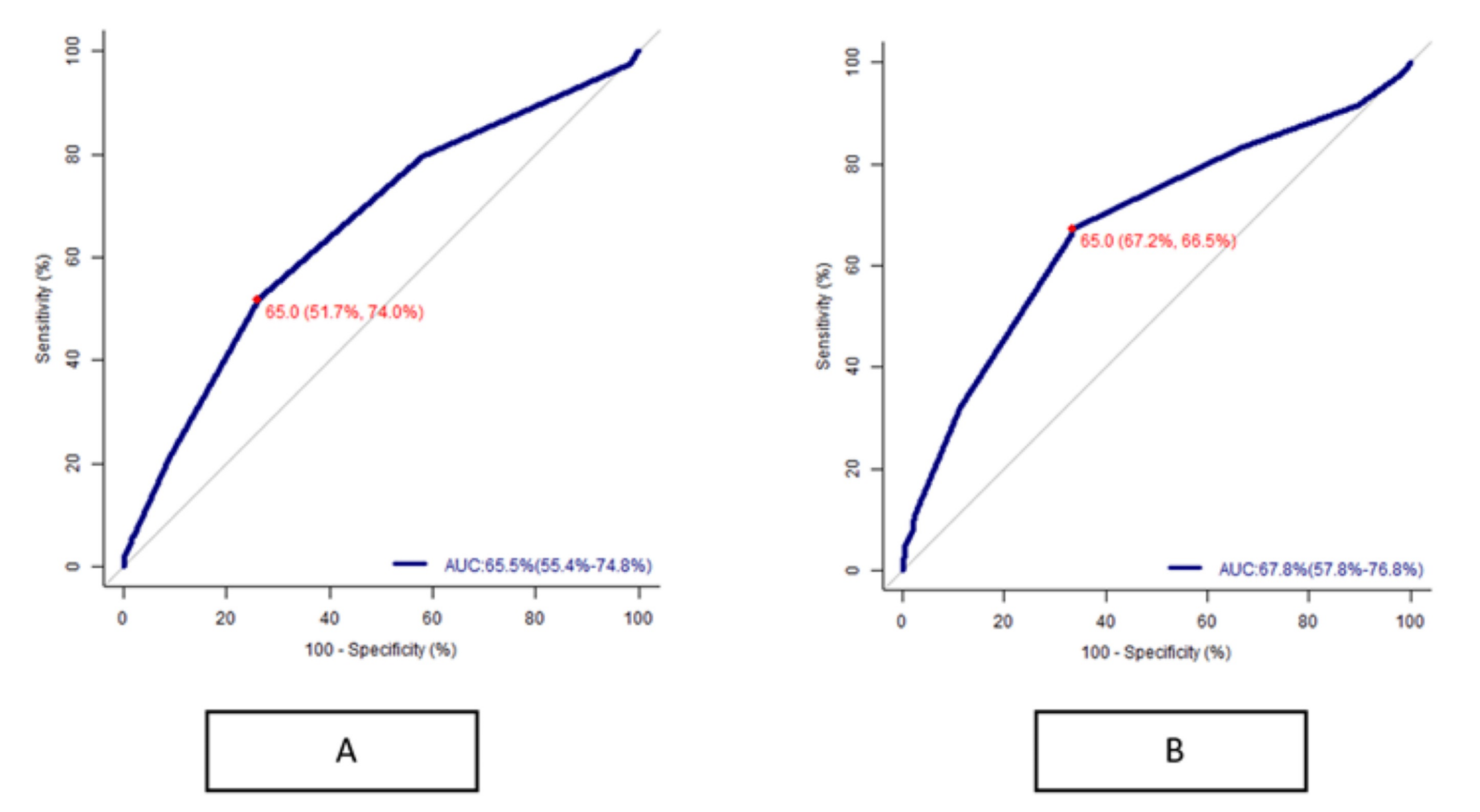
The ROC curve of peak nasal inspiratory flow for allergic rhinitis (A) and blocker-variety allergic rhinitis (B) ROC: Receiver operating characteristic, AUC: Area under the curve

Receiver operating characteristic (ROC) curve analysis was conducted to determine a diagnostic cutoff for allergic rhinitis, identifying an optimal PNIF threshold of 65 L/min, as shown in Figure 2. At this cutoff, sensitivity was 51.7% and specificity 74%. The area under the curve (AUC) reached 65.5% (95% CI: 55.4%-74.8%), and Youden’s index was calculated as 0.257.

The diagnostic utility of PNIF for each age group in both allergic rhinitis and blocker categories is shown in Table 2. From a diagnostic perspective, a PNIF value below 65 L/min increased the likelihood of detecting allergic rhinitis by 1.98 times (positive likelihood ratio), whereas values above this threshold reduced the probability by 0.5 times (negative likelihood ratio). Overall diagnostic accuracy was 58.1%.

**Table 2:** Diagnostic utility of PNIF in various age groups with allergic rhinitis PNIF: Peak nasal inspiratory flowmeter, AUC: Area under the curve, PPV: Positive predictive value, NPV: Negative predictive value

Variable	Age group	PNIF cut-off (AUC (95% CI))	Diagnostic accuracy	Sensitivity (95% CI)	Specificity (95% CI)	PPV (95% CI)	NPV(95% CI)
Allergic rhinitis			58.1%	0.517 (0.461, 0.573)	0.74 (0.681, 0.793)	0.72 (0.657, 0.777)	0.543 (0.488, 0.596)
	Six to nine years	60 (0.637 (0.534,0.731))	60.1%	0.337 (0.244, 0.439)	0.854 (0.771, 0.916)	0.688 (0.537, 0.813)	0.575 (0.493, 0.655)
	10 to 12 years	65 (0.704 (0.605,0.791))	64.19%	0.51 (0.409, 0.61)	0.867 (0.754, 0.941)	0.867 (0.754, 0.941)	0.51 (0.409, 0.61)
	13 to 17 years	70 (0.73 ( 0.634,0.816))	58.68%	0.357 (0.274, 0.447)	0.92 (0.841, 0.967)	0.865 (0.742, 0.944)	0.497 (0.417, 0.577)
Allergic rhinitis (blocker variety)			60.0%	0.672 (0.58, 0.756)	0.665 (0.62, 0.709)	0.345 (0.284, 0.41)	0.886 (0.847, 0.917)
	Six to nine years	80 (0.379 (0.284,0.482))	24.87%	0.861 (0.705, 0.953)	0.115 (0.071, 0.174)	0.175 (0.122, 0.239)	0.792 (0.578, 0.929)
	10 to 12 years	65 (0.70 (0.60,0.788))	71.60%	0.625 (0.485, 0.751)	0.764 (0.672, 0.841)	0.583 (0.449, 0.709)	0.794 (0.703, 0.868)
	13 to 17 years	60 (0.76 (0.668, 0.843))	84.50%	0.421 (0.203, 0.665)	0.887 (0.833, 0.928)	0.267 (0.123, 0.459)	0.94 (0.895, 0.97)

Subgroup analyses revealed age-dependent variations in diagnostic performance. The PNIF cut-off provided robust diagnostic accuracy for allergic rhinitis in the six-to-nine-year and 13-to-17-year age groups. For the blocker phenotype of allergic rhinitis, significant diagnostic utility was observed among children older than 10 years. Statistically significant associations were noted between age group and allergic rhinitis, as well as the blocker variety. No significant associations were identified between gender and either allergic rhinitis or its blocker form.

## Discussion

This study delineates normative reference values and introduces a clinical nomogram for PNIF among Indian children aged six to 17 years. Age emerged as the only statistically significant predictor of PNIF, whereas gender and BMI did not exert a notable influence. There was a discernible linear progression in PNIF values across age groups, consistent with physiological expectations that airway growth is associated with increased functional flow.

The mean PNIF values reported herein are consistent with those documented previously in an Indian study among children aged six to 12 years (53-85 L/min), supporting external validity within the Indian demographic. Conversely, substantial differences were observed compared with international data; mean PNIF values in a Greek population were considerably higher than those in this cohort. This divergence highlights the need for population-specific normative datasets; using Caucasian reference ranges, such as Greek data, may lead to misclassification of Indian children due to their generally smaller anthropometric parameters.

No significant gender-based difference in PNIF was identified, which contrasts with findings from Brazilian studies, in which boys consistently achieved higher flows [8], yet aligns with Dutch data [7]. The absence of sexual dimorphism in this sample may indicate later or less pronounced respiratory mechanical differentiation in the Indian population or reflect limitations in capturing older adolescent data.

A PNIF threshold of 65 L/min was defined to diagnose allergic rhinitis, with modest diagnostic accuracy in the six to nine and 13 to 17 years age bands. However, for the blocker phenotype, characterized by predominant nasal obstruction, diagnostic reliability was robust only in children above 10 years. Notably, in the six-to-nine-year blocker group, the low AUC (0.379) likely reflects biomechanical limitations inherent in younger children, in which flexible nasal cartilage may paradoxically reduce inspiratory flow during forceful effort.

Clinical identification of the 'blocker' phenotype is particularly important, as 47% of children with allergic rhinitis in this study were classified in this category. Existing prescription trends favor oral antihistamines, which are suboptimal for obstruction; thus, objective identification of blocker variety can aid optimization of therapy with prioritization of intranasal corticosteroids as first-line therapy.

A key strength of this research lies in its establishment of an age-stratified, culturally and biologically appropriate baseline nomogram for PNIF in the Indian pediatric population. Nonetheless, the cross-sectional nature restricts the insight into longitudinal growth trajectories. The hospital outpatient sample limits normative generalizability compared to a community-based survey, and the absence of a rigorously defined healthy control group further constrains interpretation.

The regression model’s adjusted R2 of 0.230 indicates that age, gender, and BMI account for less than one-quarter of the variability in PNIF, suggesting substantial influence from unmeasured factors. Thus, PNIF should not be used as a standalone diagnostic test but rather as an adjunctive objective measure to support clinical diagnosis. Clinicians should utilize the nomogram as a guide rather than an absolute predictor for individual patients. The inherent measurement precision of the PNIF device (±10% or ±10 L/min) imposes a ceiling on reproducibility that is device-specific and should be recognized when applying the nomogram or cut-off values to individual patient assessment.

## Conclusions

Our study strived to standardize the nomogram for PNIF for Indian children. Using a defined clinical threshold of 65 L/min, PNIF can serve as a viable modality for determining allergic rhinitis and identifying predominantly obstructive nasal phenotypes (blocker variety). For allergic rhinitis, it demonstrates moderate diagnostic accuracy specifically within the six to nine and 13 to 17 years age cohorts. Additionally, it exhibits clinical utility in differentiating nasal obstruction patterns in children over 10 years of age. However, it is imperative that PNIF not be relied upon as an isolated diagnostic modality. Rather, it must be integrated as an adjunctive, objective metric to corroborate comprehensive clinical evaluations. Furthermore, clinicians should interpret these nomogram values as population-specific epidemiological estimates and avoid misapplying them as generalizable normative baselines.
